# How can we maximise the benefits of smoke-free prisons? Decision analytic model to predict potential impacts on public health

**DOI:** 10.1186/s12889-026-26714-9

**Published:** 2026-02-18

**Authors:** Nicola McMeekin, Ashley Brown, Catherine Best, Evangelia Demou, Alastair H. Leyland, Linda Bauld, Nancy Loucks, Jill P. Pell, Sean Semple, Emily J. Tweed, Clair Woods-Brown, Kate Hunt, Kathleen A. Boyd

**Affiliations:** 1 Health Economics and Health Technology Assessment, School of Health and Wellbeing, University of Glasgow, Clarice Pears Building, 90 Byres Road, Glasgow, G12 8TB UK; 2 Institute for Social Marketing and Health, University of Stirling, Pathfoot Building, Stirling, FK9 4LA UK; 3 MRC/CSO Social and Public Health Sciences Unit, School of Health and Wellbeing, University of Glasgow, Clarice Pears Building, 90 Byres Road, Glasgow, G12 8TB UK; 4 Centre for Population Health Sciences, Usher Institute, University of Edinburgh, Usher Building, 5-7 Little France Road, Edinburgh BioQuarter, Edinburgh, EH16 4UX UK; 5 Families Outside, 17 Gayfield Square, Edinburgh, EH1 3NX UK; 6 Public Health, School of Health and Wellbeing, University of Glasgow, Clarice Pears Building, 90 Byres Road, Glasgow, G12 8TB UK; 7 Ayrshire and Arran NHS, Ailsa Hospital, Dalmellington Road, Ayr, KA6 6DX UK

**Keywords:** Cost-utility analysis, Prison, Smoking cessation, Tobacco, Released people, Families, Decision analytic model, Smokefree prison

## Abstract

**Introduction:**

Tobacco smoking prevalence remains high in disadvantaged populations such as people in prison. Smokefree prisons protect health, however around 90% of people who smoke pre-prison, relapse to smoking shortly after release. If people released from smokefree prisons maintain smoking abstinence this could benefit their health and finances. Knock-on effects of smoking relapse on families could also be avoided. Offering an intervention to reduce relapse to smoking on release has the potential to benefit released people and their families.

This study assesses potential costs and outcomes for released people and their families, of introducing a smokefree prison policy and an intervention to reduce post-release smoking relapse.

**Methods:**

Based on the smoking/vaping status of released people we modelled the impact, on costs and outcomes, of four scenarios. We modelled scenarios which varied across two dimensions: (1) whether people were/were not permitted to vape in smokefree prisons, and (2) whether a smoking cessation intervention was offered/was not offered in smokefree prisons. The scenarios reflect different combinations of these factors. We estimated costs and outcomes (benefits) for released people, their partners and children over a lifetime. We included personal costs (vaping and smoking), healthcare and intervention costs, and outcomes included quality of life.

**Results:**

For released people, results indicated that not permitting vaping in prison was less costly and more beneficial than when vaping was permitted. Offering a smoking cessation intervention to released people was less costly than not offering a smoking cessation intervention, irrespective of whether vaping was permitted or not. However, whilst offering a smoking cessation intervention was beneficial when vaping was permitted in prison, results are uncertain for the benefits of offering a smoking cessation intervention when vaping is not permitted in prison. Sensitivity analyses indicate uncertainty and show that changing the values for vaping prevalence and smoking relapse rates would change these results.

For both partner and child (ren), costs were higher and quality of life lower for those living with released people who relapse to smoking compared to those who vape or neither smoke nor vape.

**Interpretation:**

Targeted support for smoking cessation interventions to improve health outcomes for people released from smokefree prison and their families can ultimately contribute to broader public health improvements and improve health in a priority group. There is a need for greater evidence in this area to inform future modelling, particularly on relapse to smoking on release and the long-term effects of vaping. Results indicate uncertainty about the overall value of permitting vaping in smokefree prisons; wider factors associated with not allowing vaping in prisons would need to be assessed in future work. Study findings enhance understanding of the potential cost-effectiveness of smokefree prison policy, highlight uncertainty in some model inputs, and can inform decisions about how value could be maximised.

**Supplementary Information:**

The online version contains supplementary material available at 10.1186/s12889-026-26714-9.

## Introduction

Whilst smoking prevalence overall is decreasing, smoking rates and associated smoking related illness, and exposures to second-hand smoke (SHS), remain relatively high in disadvantaged populations [1, 2], contributing to inequalities in health. Most people in prison are from lower socioeconomic backgrounds, and historically smoking levels in prisons have been high [3, 4]. In Scotland, for example, smoking prevalence among the prison population was 68% in 2017 [5]. A smokefree prison policy was introduced in Scotland in November 2018: no-one (including staff, people in prison and visitors) can now smoke in indoor or outdoor areas of all prisons. Evidence suggests that the smokefree prison policy in Scotland effectively eliminated tobacco use and exposure to SHS in prisons [6]. Research shows that total smokefree prison policies, like Scotland’s policy, have reduced cardiovascular disease risk among people in prison who smoked prior to imprisonment [7, 8], and are associated with a 9% reduction in smoking related deaths in prison [9].

As part of the transition to smokefree prisons in Scotland, rules were changed to allow people in prison to vape, with 60% reporting vaping in 2019 in Scottish prisons [10]. The UK National Institute for Health and Care Excellence (NICE) supports vaping for smoking cessation [11], as evidence to-date suggests vaping is likely to be less harmful than smoking. There is currently insufficient evidence to quantify long-term risks of vaping [12–14].

If people who smoked prior to imprisonment in a smokefree prison were able to maintain abstinence post-release, there could be considerable health and financial benefits for individuals, and reduced pressures on healthcare services and spending. Studies of prison systems which are smokefree and *do not* allow vaping, consistently find a rapid and high return to smoking post-release [15]. This highlights a lost opportunity to address a leading cause of inequality in morbidity and mortality; evidence shows that disadvantaged groups are less likely to successfully quit smoking despite being motivated to quit [16]. To date, there is no evidence on smoking relapse rates among people released from smokefree prison systems which *do* allow vaping [15]. On one hand, switching from smoking to vaping in prison could decrease the likelihood of post-release smoking relapse by providing a potentially cheaper alternative. On the other hand, people who vape in prison may relapse to smoking post-release at a similar or even higher rate due to maintenance of nicotine dependence. Dual vaping and smoking might also increase post-release with little if any benefit to health.

Secondary negative impacts of smoking on family members could also be averted if those who smoked prior to imprisonment in a smokefree prison remained abstinent post-release. In Scotland, over 20,000 children are affected by parental imprisonment every year [17]. Having a smoking parent increases the likelihood of children being exposed to SHS in homes and experiencing smoking-related diseases [18, 19]. Children living with people who smoke are also more likely to start smoking themselves [20]. Smoking disproportionately impacts the finances of families living on low incomes and exacerbates child poverty [21, 22]. Imprisonment can place considerable (additional) financial strain on families. Respondents to a recent survey reported spending a third of their monthly income on a family member in prison, and around a half of their monthly income to support a family member after release [23]. Reducing or eliminating smoking costs could therefore make a substantial difference to the lives of families affected by imprisonment.

The multi-phase, multi-method Tobacco in Prisons Study (TIPS) assessed the impact of introducing smokefree prison policy in Scotland [24]. An economic evaluation found that introducing the policy was cost-effective for people in prison and staff over a lifetime [25], although limited data were available to inform some estimates (e.g. post-release relapse rates). We originally planned to try to address these evidence gaps in a follow-up study (Staying smokefree: Maximising the public health benefits of smokefree prisons – TIPS2) by updating the lifetime model for people in prison and, for the first time, modelling the costs and outcomes of smokefree prison policies for families affected by imprisonment. However, the effects of the COVID-19 pandemic in prisons, combined with other factors, prevented this. The current study therefore assesses the potential costs and outcomes of smokefree prison policy for people previously imprisoned and their families under a range of plausible scenarios. Findings from the study can increase understanding of the potential cost-effectiveness of smokefree prison policy and inform decisions about how value could be maximised.

## Methods

### Overview

A modelling approach was taken to simulate the plausible scenarios. Costs and outcomes were modelled for people recently released from a smokefree prison (hereafter referred to as ‘released people/person’), and their partners and children (from age 15) in a Scottish household setting. Costs and outcomes were estimated over a lifetime in yearly cycles.

Due to limitations imposed by the pandemic and in the global evidence base, as noted above, we modelled a scenario, in which a hypothetical intervention to support smoking cessation and prevent relapse to smoking post-release (hereafter referred to as the ‘intervention’) was offered in smokefree prisons.

Methods and results are presented in line with established guidance [26]. The Methods below focus on contextual explanations, with technical details available in Supplementary Material and model input parameters in Supplementary material Table S1.

### Model: released people

For released people we modelled four scenarios dependent on two factors: (a) whether vaping is permitted in smokefree prisons, and (b) whether the intervention is offered (Table 1).

**Table 1:** Model scenarios

**Scenario 1**	**Scenario 3**
Vaping-permitted	Vaping-permitted
No intervention offered	Intervention offered
**Scenario 2**	**Scenario 4**
No vaping permitted	No vaping permitted
No intervention offered	Intervention offered

To evaluate cost-effectiveness for released people we compared three pairs of these scenarios, described in detail in ‘Analysis’ sub-section below.

**Fig. 1:**
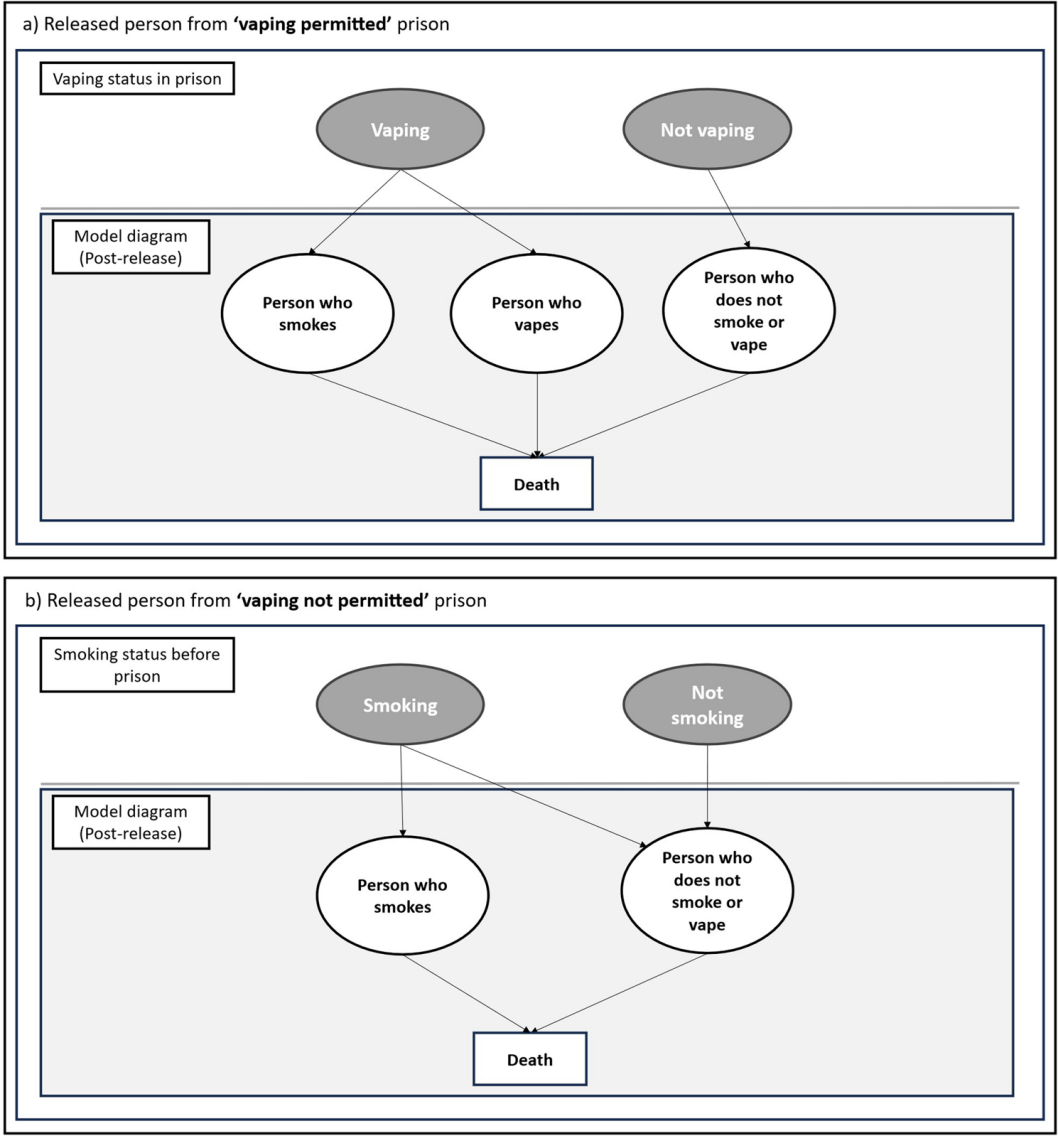
(**a** when person is released from 'vaping permitted' prison, **b** when person is released from 'vaping not permitted' prison)

To enable these comparisons two models were used based on the smokefree policy of prison (Fig. 1). Assumptions about smoking/vaping status for released people are based on (a) vaping status in ‘vaping-permitted’ prisons, or (b) smoking status prior to imprisonment in ‘no vaping permitted’ prisons.

In the ‘vaping-permitted’ scenarios (a), we assume people who vape in prison will either smoke or vape post-release, and people who do not vape in prison will not smoke or vape. In the ‘no vaping permitted’ scenarios (b), we assume that people who smoked prior to imprisonment will either relapse to smoking or will not smoke or vape. Both models are run for a lifetime.

There is limited evidence on relapse to smoking post-release from smokefree prisons (and none from those where vaping is permitted); we applied the best avaliable evidence from Australian smokefree prisons where the prison context has similarities to that of the UK (see Supplementary Materials for more detail).

### Smoking cessation intervention

We assumed that the intervention was available to people who smoked prior to imprisonment in the ‘no vaping permitted’ scenarios, and to people who vape in ‘vaping-permitted’ prisons, and that it would be offered around the time of release. The cost and effectiveness of the intervention is applied in the model in the first year after release. We based assumptions about intervention uptake on data on the proportion of people in prison who wish to give up smoking [5].

### Model: partner/child

**Fig. 2:**
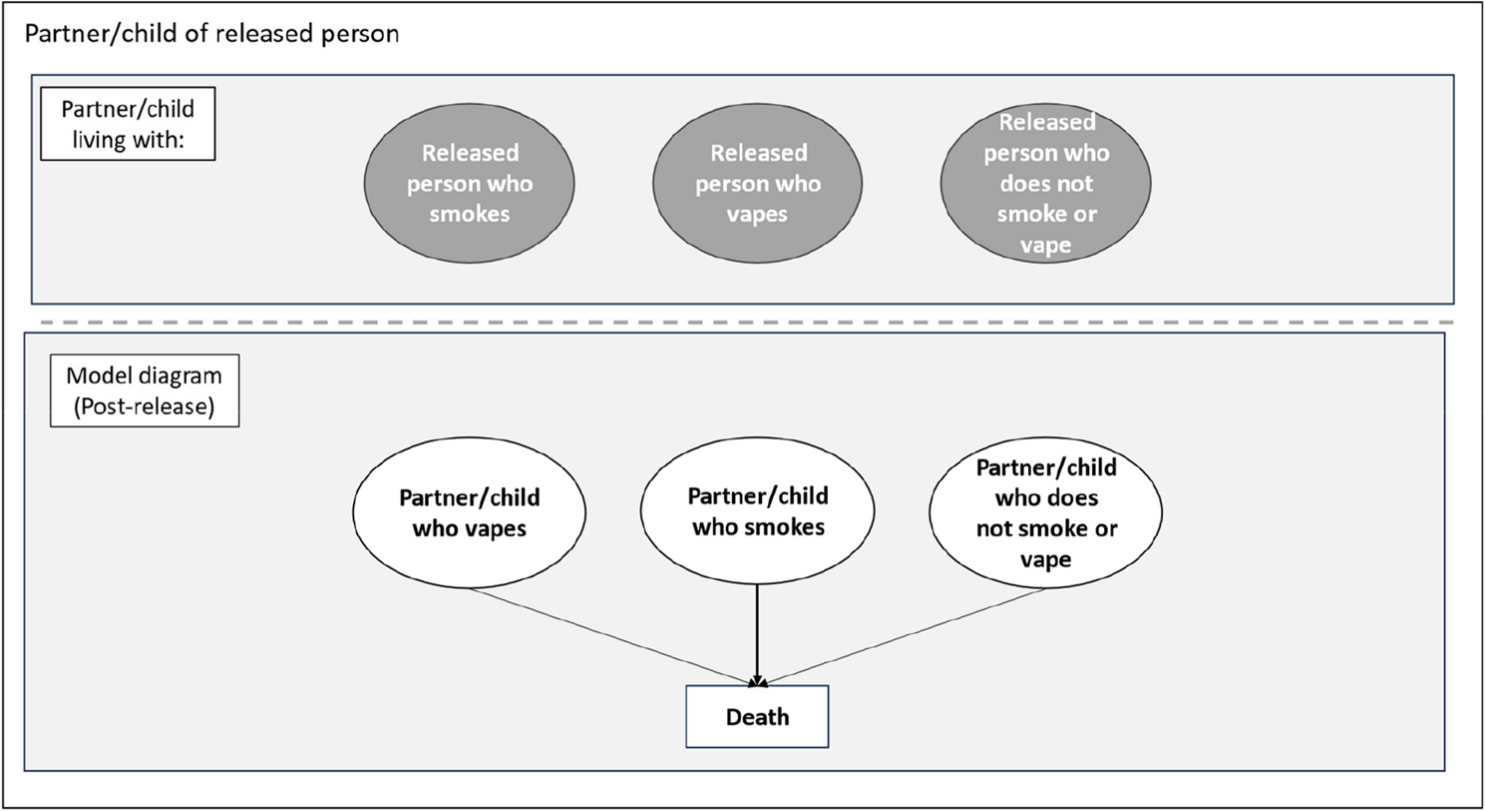
Model structure - partner/child

For the partner/child model we assessed three scenarios; living with released people who smoke, vape or do not vape or smoke (Fig. 2).

Given that around 95% of the prison population are men and more come from the least affluent circumstances, we applied an assumption that partners are female (in the absence of robust information on other partnerships/household circumstances in those leaving prison) and hence applied general population smoking and vaping rates for women living in the most deprived neighbourhoods to simulate partner smoking status. We assumed that child uptake of smoking or vaping is dependent on the vaping or smoking status of released people. Partners and children are allocated to smoking status (vape, smoke, or do not vape or smoke) based on these rates. This model is run for a lifetime until the model participants are in the ‘death’ state.

### Morbidity

Current evidence shows that vaping is less harmful than smoking, however vaping is unlikely to be risk-free, and current evidence (at the time of the modelling) on vaping harms is mostly limited to studies of less than 12-months, creating challenges for this type of long-term model [27]. Our model reflects gradients of harm, with the basic premise that smoking is most harmful, vaping carries some intermediate health risk, and not smoking or vaping carries least risk. For people in all models who smoke we applied smoking related disease prevalence, for people who vape we applied vaping harms (acute bronchitis incidence), and for partners who live with released people who smoke we applied harms for secondhand smoke exposure. No harms are applied to people who do not smoke/vape and do not live with a person who smokes.

### Mortality

General population mortality rates were applied to all model populations, with increased risk applied to those who smoked, were exposed to secondhand smoke and who had been released from prison.

### Costs

The costs included in the models include three types of resource use, i.e. healthcare (smoking related disease treatment and harms from vaping), personal (smoking and vaping products), and intervention (released people only). All costs are applied over the entire time horizon of the model (lifetime).

### Outcomes

Outcomes include quality-of-life, life-years (number of years survived) and smoking/vaping status. Quality-of-life is presented as a quality-adjusted life-year (QALY), which is a combination of quality-of-life (health utilities) and length of life (life-years) given as a single combined outcome. The scale for QALYs is from zero to one, where ‘0’ represents death and ‘1’ represents full health.

### Analysis

For released people a cost-utility analysis was conducted; this type of analysis assesses the difference in costs and QALYs when comparing different scenarios. The three comparisons included in this analysis were: (i) vaping permitted in smokefree prisons vs. no vaping permitted in smokefree prisons, with no intervention offered; (ii) no intervention offered vs. intervention offered (in vaping-permitted smokefree prisons), and (iii) no intervention offered vs. intervention offered (in no vaping permitted smokefree prisons). Mean costs and outcomes are presented with 95% confidence intervals (95%CI). Incremental results are used to estimate incremental cost-effective ratios (ICERs), where the difference in costs between the comparators is divided by the difference in QALYs. Incremental net monetary benefit (iNMB) is calculated using a willingness-to-pay threshold of £20,000. iNMB combines difference in cost and QALYs with willingness-to-pay threshold, with results above zero indicative of cost-effectiveness.

Partner and child results (mean costs and outcomes) are presented for three household scenarios where the partner/child lives with a released person who (1) smokes, or (2) vapes or (3) neither smokes nor vapes.

Uncertainty in the model parameters was assessed with probabilistic sensitivity analysis using best practice methods [28]. Results were used to estimate 95%CI around costs and QALYs and illustrated in a cost-effectiveness plane (presented in Supplementary Material).

### Sensitivity analyses

To understand the potential of smokefree policies and due to uncertainty in the parameters, several sensitivity analyses were conducted where key model parameters were varied to assess the impact on results. For released people, the parameters varied were: vaping prevalence in prison; relapse to smoking on release; harms from vaping; uptake of the intervention; and intervention intensity (cost and quit rate). For the child model, best-case and worst-case scenarios of uptake of smoking in children (dependent on parent smoking status) were applied. Results are illustrated in a tornado diagram (released people) and table (released people and child) using iNMB.

## Results

### Released people

Total costs ranged from £26,880 (95%CI £22,392, £32,169) for Scenario 4 (no vaping permitted, intervention offered), to £31,347 (95%CI £26,451, £37,271) for Scenario 1 (vaping-permitted, no intervention offered) (Table 2). The largest proportion of total costs, in all scenarios, were for personal (smoking and vaping) costs to released people, accounting for around 78% of total costs. For the vaping-permitted scenarios, 97% of personal costs were for smoking products and 3% vape products; for no vaping permitted scenarios all personal costs were for smoking products (relative price for smoking/vaping products was based on the price in the community in 2022, see Supplementary Material for more details). Lifetime total costs for released people are lowest when vaping is not permitted in a smokefree prison and the intervention is offered. Healthcare costs made up around 22% of total costs and intervention costs were less than 1% (£40). Mean intervention costs (approximately £40) are lower than the actual intervention cost (£112) as not all released people are offered the intervention (only those who smoke prior to imprisonment in a no vaping prison and those who vape in a vaping-permitted prison), we also assume not everyone who is offered the intervention will engage with it.

**Table 2:** Results – released person

	No intervention				Intervention			
	Vaping permitted in smokefree prison (Scenario 1)		No vaping permitted in smokefree prison (Scenario 2)		Vaping permitted in smokefree prison (Scenario 3)		No vaping permitted in smokefree prison (Scenario 4)	
	Mean	95% CI	Mean	95% CI	Mean	95% CI	Mean	95% CI
Costs (discounted)								
Healthcare	£ 6,673	£5,930 to £7,533	£6,137	£5,467 to £6,957	£6,393	£5,681 to £7,220	£6,080	£5,423 to £6,877
Personal	£ 24,674 (3% vape, 97% tobacco)	£19,870 to £30,175	£21,446 (all tobacco)	£16,972 to £26,449	£23,889 (3% vape, 97% tobacco)	£19,238 to £29,215	£20,764 (all tobacco)	£16,432 to £25,608
Intervention	-	£0 to £0	-	£0 to £0	£43	£43 to £43	£36	£33 to £37
Total	£ 31,347	£26,451 to £37,271	£27,584	£22,953 to £33,039	£30,325	£25,585 to £36,054	£26,880	£22,392to £32,169
Life-years (discounted)	17.20	16.89 to 17.48	17.39	17.11 to 17.66	17.23	16.94 to 17.51	17.42	17.16 to 17.68
QALYs (discounted)	13.54	13.22 to 13.81	13.79	13.50 to 14.06	13.58	13.28 to 13.85	13.82	13.54 to 14.06
Prevalence								
Non-nicotine	24%		37%		24%		37%	
Person who smokes	70%		63%		68%		61%	
Person who used to smoke	0%		0%		2%		2%	
Person who vapes	6%		0%		6%		0%	
Person who used to vape	0%		0%		0%		0%	

Cost-utility analysis – comparing Scenarios						
	Permitting vaping v. not permitting vaping with no intervention in smokefree prison (Comparison 1)		Vaping permitted in smokefree prison (Comparison 2)		No vaping permitted in smokefree prison (Comparison 3)	
	Vaping permitted (Scenario 1)	No vaping permitted (Scenario 2)	No intervention (Scenario 1)	Intervention (Scenario 3)	No intervention (Scenario 2)	Intervention (Scenario 4)
Total costs (discounted)	£31,347 (95% CI £26,451 to £37,271)	£27,584 (95% CI £22,953 to £33,039)	£31,347 (95% CI £26,451 to £37,271)	£30,325 (95% CI £25,585 to £36,054)	£27,584 (95% CI £22,953 to £33,039)	£26,880 (95% CI £22,392 to £32,169)
Total QALYs (discounted)	13.54 (95% CI 13.22 to 13.81)	13.79 (95% CI 13.50 to 14.06)	13.54 (95% CI 13.22 to 13.81)	13.58 (95% CI 13.28 to 13.85)	13.79 (95% CI 13.50 to 14.06)	13.82 (95% CI 13.54 to 14.06)
Incremental (costs and QALYs)	£3,764 (95% CI £2,715 to £5,125)	-0.252 (95% CI -0.355 to -0.17)	£1,022 (95% CI £866 to £1,209)	-0.044 (95% CI -0.056 to -0.033)	£704 (95% CI £561 to 871)	-0.037 (95% CI -0.126 to 0.045)
Cost per QALY	Dominated (more costly and less effective)		Dominated (more costly and less effective)		Dominated (more costly and less effective)	
iNMB	-£8,798		-£1,911		-£1,437	

*iNMB *Incremental net monetary benefits, *QALY *Quality adjusted life-year

Released people previously imprisoned in a no vaping permitted smokefree prison *and* offered the intervention have slightly better outcomes (life-years and QALYs gained), compared to released people in the other three scenarios. Life-years ranged from 17.20 (95%CI 16.89, 17.48) (Scenario 1) to 17.42 (95%CI 17.16, 17.68) (Scenario 4), and QALYs ranged from 13.54 (95%CI 13.22, 13.81) (Scenario 1) to 13.82 (95%CI 13.54, 14.06) (Scenario 4). Whilst the differences between these outcomes at an individual level are small, when applied to the population of people released they are significant, particularly from a public health perspective.

The cost-utility analysis results show that in Comparison 1 the scenario permitting vaping is more costly (£3,764 (95%CI £2,715, £5,125)) and less effective (QALYs − 0.252 (95%CI -0.355, -0.170)) than not permitting vaping. In Comparison 2, when vaping is permitted, not offering an intervention is more costly (£1,022 (95%CI £866, £1,209)) and less effective (QALYs − 0.044 (95%CI -0.056, -0.033)) than offering an intervention. In Comparison 3 when vaping is not permitted, not offering an intervention is more costly (£704 (95%CI £561, £871)) and less effective (QALYs − 0.037 (95%CI -0.126, 0.045)) than offering an intervention. These latter QALY outcome results are uncertain as the 95% CI crosses zero.

The incremental Net Monetary Benefit analysis reflects these results; not permitting vaping is more cost-effective than permitting vaping, and offering an intervention is more cost-effective than not offering an intervention.

### Partner and child

The largest proportion of costs for partners were personal costs (smoking and vaping) for all three scenarios (Table 3), around two thirds of total costs. Personal costs were split 87% for smoking products and 13% for vaping products. Healthcare costs were around one third of total costs and slightly higher for partners living with a released person who smokes due to SHS exposure (£5,547 (95%CI £5,053, £6,090) compared to £5,295 (95%CI £4,821, £5,809) where the released person vapes or neither smokes nor vapes. There is very little difference in life-years and QALYs between different scenarios: 22.15 (living with released person who smokes) v. 22.16 (living with released person who vapes or neither smokes nor vapes) and 17.54 (living with released person who smokes) v. 17.82 (living with released person who vapes or neither smokes nor vapes) respectively.

**Table 3:** Results - parent and child

	Living with person released from prison who does not smoke or vape		Living with person released from prison who smokes		Living with person released from prison who vapes	
	Mean	95% CI	Mean	95% CI	Mean	95% CI
Partner						
Costs (discounted)						
Healthcare	£5,295	£4,841 to £5,809	£5,547	£5,053 to £6,090	£5,295	£4,813 to £5,785
Personal	£10,617 (13% vape, 87% tobacco)	£8,157 to £13,610	£10,617 (13% vape, 87% tobacco)	£8,157 to £13,610	£10,617 (13% vape, 87% tobacco)	£8,157 to £13,610
Intervention	-		-		-	
Total	£15,912	£13,212 to £19,018	£16,164	£13,449 to £19,280	£15,912	£13,212 to £19,018
Life-years (discounted)	22.16	22.06 to 22.25	22.15	22.04 to 22.24	22.16	22.06 to 22.25
QALYs (discounted)	17.82	17.61 to 18.01	17.54	17.36 to 17.70	17.82	17.61 to 18.01
Prevalence						
Non-nicotine	63%		0%		63%	
Non-nicotine (exposed to SHS	0%		63%		0%	
Person who smokes	21%		21%		21%	
Person who used to smoke	0%		0%		0%	
Person who vapes	10%		10%		10%	
Person who used to vape	0%		0%		0%	
Child						
Costs (discounted)						
Healthcare	£2,800	£2,563 to £3,067	£3,596	£3,264 to £3,966	£3,282	£2,989 to £3,607
Personal	£10,312 (22% vape, 77% tobacco)	£8,542 to £12,495	£24,875 (17% vape, 83% tobacco)	£20,472 to £30,386	£19,022 (17% vape, 83% tobacco)	£15,671 to £23,198
Intervention						
Total	£13,113	£11,268 to £15,300	£28,471	£24,009 to £34,046	£22,304	£18,876 to £26,552
Life-years (discounted)	25.69	25.66 to 25.70	25.49	25.42 to 25.54	25.56	25.51 to 25.60
QALYs (discounted)	22.02	21.89 to 22.13	21.41	21.18 to 21.62	21.66	21.47 to 21.83
Prevalence						
Non-nicotine	70%		33%		49%	
Non-nicotine (exposed to SHS)	0%		0%		0%	
Person who smokes	15%		39%		30%	
Person who used to smoke	0%		0%		0%	
Person who vapes	15%		27%		22%	
Person who used to vape	0%		0%		0%	

*CI* Confidence interval, *SHS *Second-hand smoke, *QALY *Quality adjusted life-year

The highest costs for children (from age 15) were personal costs in all scenarios, ranging from £10,312 (95%CI £8,542, £12,495) for living with a released person who does not smoke or vape to £24,875 (95%CI £20,472, £30,386) for living with a released person who smokes. For children living with a released person who does not smoke or vape personal costs were split 77% for smoking products and 23% for vaping products, and for living with a released person who smokes or vapes split 83% for smoking products and 17% for vaping products. Healthcare costs were higher for children living with a released person who smokes (£3,596 (95%CI £3,264, £3,966)), compared to children living with a released person who neither smokes nor vapes (£2,800 (95%CI £2,563, £3,067)). QALY outcomes, ranged from 21.41 (95%CI 21.18, 21.62) for children of released parents who smoke, to 22.02 (95%CI 21.89, 22.13) for children of released parents who neither smoke nor vape. The prevalence of children who smoke [or vape] ranges from 15% [15%] for children who live with released people who neither smoke nor vape, to 39% [27%] for children who live with released people who smoke.

### Sensitivity analyses

**Fig. 3:**
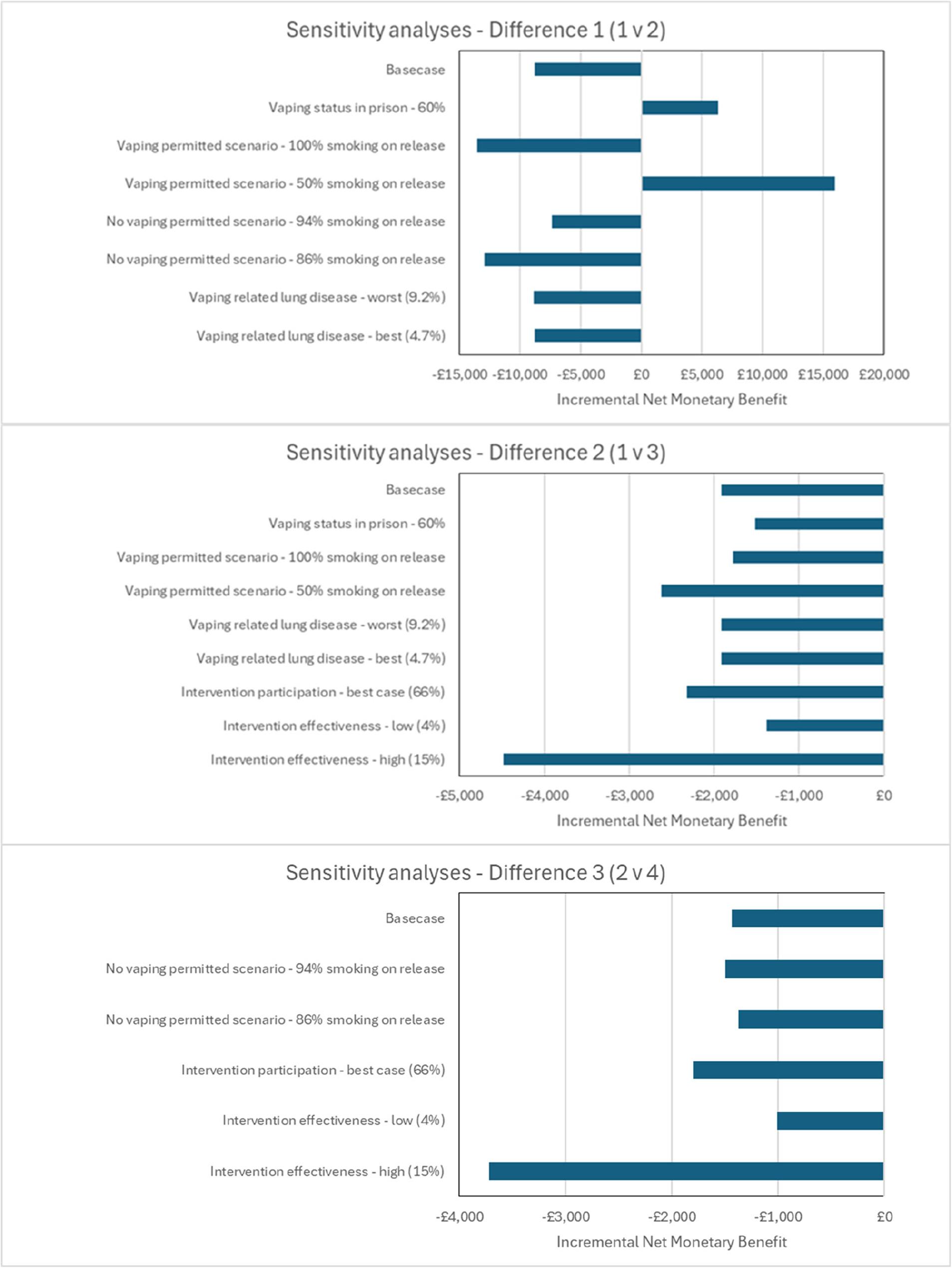
Sensitivity results - released people

Results are presented in Fig. 3 and Supplementary Material Table S2 for released people. In Comparison 1 (comparing vaping-permitted v no vaping-permitted smokefree prisons with no intervention offered) permitting vaping becomes a cost-effective scenario (less costly and more beneficial) if (1) the vaping rate in prison is lowered to 60% or (2) the smoking relapse rate is lowered to 50% in vaping-permitted prisons (reversing basecase results).

In Comparison 2 (comparing vaping-permitted smokefree prisons with/without an intervention), offering the intervention remains cost-effective for all changes in model parameters. The two parameter changes with the biggest positive impact are assuming (1) 50% of people who vape in prison relapse to smoking post-release, and (2) increasing the effectiveness of the intervention.

In Comparison 3 (comparing vaping not permitted smokefree prisons with/without an intervention) all parameter changes resulted in a cost-effective result. The two parameters with the biggest positive impact on results (making the intervention more cost-effective than basecase) are: (a) increasing intervention engagement to 66% and (b) increasing the effectiveness of the intervention.

In the children’s model sensitivity analyses (Supplementary Material Table S3), increasing the uptake of smoking/vaping for children living with a parent who smokes from 15.2% (basecase) to 22.8% (worst case scenario) has a negative impact (i.e. more costly and less beneficial). Conversely, decreasing smoking uptake to 7.6% (best case scenario) has a positive impact (i.e. less costly and more beneficial).

## Discussion

### Interpretation of results

Our baseline model suggests that not permitting vaping in a smokefree prison may result in lower costs and better outcomes for released people compared to a scenario in which vaping is permitted. However, when varying seven parameters in a sensitivity analyses two parameters − (1) lower vaping prevalence in prison, and (2) lower levels of relapse to smoking on release from vaping-permitted prisons - suggested that not permitting vaping may be more costly and less beneficial. These findings indicate uncertainty due to limited evidence for some key parameters (e.g. rates of post-release smoking relapse).

Offering an intervention around the time of release from both no vaping permitted and vaping-permitted prisons reduces future personal and healthcare costs. However there are uncertainties in the benefits depending on whether vaping is or is not permitted; in vaping-permitted prisons offering the intervention improves outcomes, but in no vaping permitted prisons the impact of the intervention on outcomes is uncertain. Sensitivity analyses comparing offering and not offering the intervention in the different vaping scenarios found that results remained cost-effective in all analyses; the variations in assumptions with the biggest impact were reducing relapse to smoking post-release and increasing engagement with the intervention and its effectiveness.

In both the partner and child models, the most costly scenario was living with released people who smoke. In the child model the negative impact of released people’s smoking status on children’s smoking status was clear, increasing personal costs and adversely impacting health outcomes. This impact on children who live with released people who smoke is of significance to healthcare providers and public health professionals. These children are more than twice as likely to smoke and spend over twice as much on smoking and vaping products as children of released people who neither smoke nor vape.

In all models there was little variation in life-year and QALY outcomes between scenarios. However, whilst these differences are small for individuals, they are large when applied at a population level, which has significance to public health professionals, healthcare providers and decision makers.

### Limitations

The biggest limitation is lack of evidence in this field: we went to considerable effort to source the most robust evidence available, but lack of reliable evidence remains a major constraint. The most substantial gap in the evidence that we found was smoking status on release from smokefree prisons where vaping is permitted; the best evidence we had for relapse to smoking on release was from a ‘no vaping permitted’ smokefree prison system in Australia which we also applied to our ‘vaping-permitted’ scenarios in the absence of any other evidence [29, 30]. Whilst we acknowledge that the policy for and population attitude towards vaping in Australia are different to the UK, the prison populations share similarities, and this prison context is thus more relevant than the general population to this analysis. For example, smoking abstinence is enforced in smokefree prisons, and so the driver for not smoking differs in this important respect between the prison and general populations. Furthermore, we do not apply any parameters to replicate the benefit of vaping as an aid to quitting. This is due to lack of evidence (to our knowledge) on the effectiveness of vaping for smoking cessation following a period of enforced smoking abstinence; this may overstate the disadvantages of vaping-permitted policies.

There is limited evidence to date on the extent of health harms from vaping. Whilst UK NICE recommends vaping for smoking cessation [11], there is emerging evidence that vaping [12–14] and exposure to second-hand aerosols from vapes [31, 32] may be harmful to health. We used a conservative approach to modelling vaping harms and simply considered the increased risks around acute bronchitis from vaping; our premise was that smoking is most harmful, not smoking or vaping is least harmful and vaping is somewhere in between. It is noteworthy that future evidence on vaping-related harms from general population studies may not be applicable to released people; evidence to date suggests that people in prison have different ways of using vapes, including heavy use and consumption of illicit substances [33, 34].

Unfortunately, due to the impact of COVID-19 on the prison system, we were unable to develop a bespoke intervention to support released prisoners to remain smoking abstinent and so had to base parameters on a hypothetical intervention. There is a small amount of evidence on intervention effectiveness (for post-smokefree prison smoking abstinence) from Australia [35–37], but this is limited to short time periods and small studies. Therefore we based our parameters on a NICE model with low, medium and high effectiveness and related costs [11] developed for a general population of people who smoke. We assume that our intervention is offered to people who do not want to relapse to smoking on release, either to people who are about to be released and/or who have recently been released.

There is a lack of evidence for living status on release; we were unable to source relevant evidence on how many people live with partners/parents/a friend/alone or homeless/temporary housing when released from custody. As a solution to this problem, we modelled partners and children separately, and readers can use results that are relevant to their needs. Assuming that the gender of all released people was male and all partners female was a simplification in the model, however we believe the impact of this simplification on results to be minimal, mainly because around 95% of people in prisoner are male.

Scarce evidence for uptake of smoking or vaping in children living with a parent who smokes or vapes resulted in us using evidence for ‘ever vapers’ and ‘ever smokers’ (sic.) [38], and we are aware that patterns of vaping in children and young people have been changing quickly in recent years [39].

As in all models we made simplifying assumptions. For example, we did not take account of reimprisonment, although we know that many released people may return to prison at a later date [40]. This makes our models conservative, as reimprisonment removes the ability to smoke tobacco during the prison stay and any intervention offered to reduce relapse to smoking would potentially be more beneficial if people released from prison are exposed to it multiple times. We did not include dual users of tobacco and vapes; there was a lack of evidence for specific harms in this population and no evidence on how many released people are dual users. We assumed that partner smoking status is not influenced by the smoking status of the released person as, again, there is no evidence in this area. Lastly, our assumption that children start in the model at age 15 likely underestimates cost savings and benefits for this cohort had the model started at a younger age for children.

People in prison are known to have a high mortality rate immediately after release [41], making it hard to separate out complexities in this population to inform a model: our simplifying assumptions are unable to fully assess the relationships between and within these complexities. Additionally, the prison population is more deprived and likely to experience higher mortality than the general population.

### Implications

Smoking continues to be a public health priority; populations with high smoking prevalence are at high risk of smoking related morbidity and mortality. These results are important to healthcare and prison sector decision makers; lower personal cost is beneficial to household spending, particularly important as most released people are from lower socioeconomic backgrounds. Improved health outcomes are important to households (including lower burden on time and travel expenses for healthcare visits and improved ability to work), to healthcare providers (lower costs and demands on services) and for society (including improved productivity). Our results emphasise the importance of providing support to remain smokefree post-release which may include support for those who wish to cut down or quit vaping whilst imprisoned in a smokefree prison where vaping is permitted. This offers potential cost-savings, both to individuals and healthcare provider, and impacts health for future generations.

This research found that offering support to remain smoking abstinent post-prison is beneficial, but there are many challenges to implementing such an intervention in this population, for example, in terms of resources, person in prison/family desire and ability to engage, reach for those with unstable living conditions and general logistics. As far as we are aware this is the first research conducted which assesses the impact of smokefree prison policies on families on release.

Implementing smokefree prisons is a complicated and multifaceted problem. Our modelling shows uncertainty about the cost-effectiveness of permitting vs. not permitting vaping in smokefree prisons due to the limited evidence base. Jurisdictions considering moving forward with smokefree prisons will need to take account of many other factors when deciding on how best to support people in prison to manage during a period of enforced smoking abstinence.

Current government policy is for the UK to become smokefree by 2030 [42]. However, the Khan Report states that, for the most deprived groups in society, this target is only likely to be met in 2044, leaving residual populations with high smoking prevalence. More resource is needed to support these populations to become smokefree [43]. By improving the health of disadvantaged groups (such as people with drug and alcohol dependence, people who are homeless and people in prison/released), the health of the general population will be improved and inequalities will decrease [44]. Rates of ill health are much higher in these disadvantaged groups, and although they are a small proportion of the population, improving their health will improve the health of the general population.

### Future research

Our previous work recommended further work to address gaps in the evidence base internationally: relapse to smoking post-release; spillover effects of smoking status on households post-release, and impacts of e-cigarette harms in the prison population [25]. This study is to our knowledge the first internationally to partially address these recommendations. A scoping review, conducted as part of the TIPS2 study, has provided a foundation for evidence on relapse rates to smoking after leaving a smokefree prison [30]. This modelling study provides initial evidence on the knock-on effects of the smoking status of released people on household members and on the post-prison period in general, as far as we are aware, the first to do so. It also provides information on the potential benefits of offering an intervention which could help to develop future interventions and policies. However, more research is needed in these areas, and in particular in identifying vaping harms in the general population and the prison population and developing effective smoking relapse prevention interventions for this population remains a priority.

## Conclusion

Offering an intervention to support released people to remain smoking abstinent has the potential to be a cost-effective approach in smokefree prisons where vaping is permitted. Sensitivity analyses show that whether permitting vaping is cost-effective or not depends on levels of vaping and smoking relapse in release; there is little evidence to inform these parameters.

## Supplementary Information

Supplementary Material 1.

## Data Availability

Data sharing is not applicable to this article as no datasets were generated or analysed during the current study. Access to the model is however available from the lead author upon reasonable request (Dr Nicola McMeekin: nicola.mcmeekin@glasgow.ac.uk).

## Abbreviations

ICER: Incremental cost-effective ratio
iNMB: Incremental net monetary benefit
NICE: National Institute for Health and Care Excellence
QALY: Quality-adjusted life-year
SHS: Second-hand smoke
TIPS: Tobacco in Prisons Study
TIPS2: Staying smokefree: Maximising the public health benefits of smokefree prisons
UK: United Kingdom
