# Supplementary material for: How can we maximise the benefits of smoke-free prisons? Decision analytic model to predict potential impacts on public health
Source: BMC Public Health. 2026 Feb 18;26:994. doi: 10.1186/s12889-026-26714-9 (PMC13019869; doi:10.1186/s12889-026-26714-9)
Supplement: Supplementary file 1 — Supplementary Material 1. [file 12889_2026_26714_MOESM1_ESM.docx]

# **How can we maximise the benefits of smoke-free prisons? Decision analytic model to predict potential impacts on public health**

Nicola McMeekin, Ashley Brown, Catherine Best, Evangelia Demou, Alastair H. Leyland, Linda Bauld, Nancy Loucks, Jill P. Pell, Sean Semple, Emily J. Tweed, Clair Woods-Brown, Kate Hunt, Kathleen A. Boyd

# SUPPLEMENTARY MATERIAL

## Overview of economic evaluation

The populations included in the evaluation were people recently released from a smokefree prison (hereafter referred to as ‘released person/people’), their partners and children (from age 15). The setting is post-prison release in a Scottish household.

The economic evaluation took a modelling approach; costs and outcomes were included from both the healthcare payer (public purse) and personal (cost to individuals) perspectives. The time horizon used was lifetime for all populations.

A discount rate of 3.5% was applied to costs and quality adjusted life-years (QALYs) in line with current recommendations from NICE(1).

## Model structures

Two models were developed using Excel (Microsoft Corporation, 2018. Microsoft Excel, Available at: [https://office.microsoft.com/excel](https://www.researchgate.net/deref/https%3A%2F%2Foffice.microsoft.com%2Fexcel?_tp=eyJjb250ZXh0Ijp7ImZpcnN0UGFnZSI6InF1ZXN0aW9uIiwicGFnZSI6InF1ZXN0aW9uIn19)), one for released people and another for their partners/children. Cycle lengths in both models were for one year and model participants accumulated costs and quality of life each cycle as they transitioned through the model over a lifetime. The models include up to four possible smoking status and health states that the model population can be allocated to, three relating to smoking/vaping (nicotine) states and one ‘Death’ state. The three nicotine states were: ‘Person who vapes’; ‘Person who smokes (tobacco)’, and ‘Person who neither smokes nor vapes’ (hereafter described as ‘nicotine-free’). The model is run for a lifetime, and each cycle, model participants either remain in their nicotine state or transition to the ‘Death’ state.

### Released people model

The structure of the released people models is primarily based on the smokefree policy of the prison: (a) vaping, and (b) no vaping (Figure 1).

In a), allocation to smoking/vaping status in the first model cycle is dependent on vaping status whilst in a ‘vaping-permitted’ prison (vaping status was calculated by CB using anonymised SPS ‘canteen’ purchasing data). We assumed that people who vape in prison will either relapse to smoking or continue vaping post-release, and people who do not vape in prison remain nicotine-free. This is a conservative approach, with those people addicted to nicotine during imprisonment continuing to be addicted on release without access to a smoking cessation intervention. As a recent scoping review found no evidence on smoking/vaping rates following release from a smokefree prison which permits vaping(2), we have applied evidence from smokefree prisons where vaping is not permitted: basecase is 92% of people who vape in prison will smoke on release and 8% will vape(3). We took this conservative approach as it is not straightforward to translate evidence on vaping status and quitting in the general population to the prison populations. Furthermore, the evidence we used from the scoping review was for a prison population in Australia, which is similar to the prison population in UK, with the prison context being more important than general population attitudes to vaping and vaping legislation.

In b), allocation to smoking/vaping status in the first model cycle is dependent on smoking status prior to imprisonment. We assumed that people who smoked prior to imprisonment either relapse to smoking or be nicotine-free. Based on the aforementioned evidence from smokefree prisons where vaping is not permitted, we applied a basecase assumption that 92% of people who smoked prior to imprisonment relapse on release, the remaining 8% were nicotine-free(3). Released people who were nicotine-free prior to imprisonment remain nicotine-free on release. We used this conservative assumption, that people addicted to nicotine pre-imprisonment will mainly return to smoking post release with a small number (8%) remaining nicotine-free.


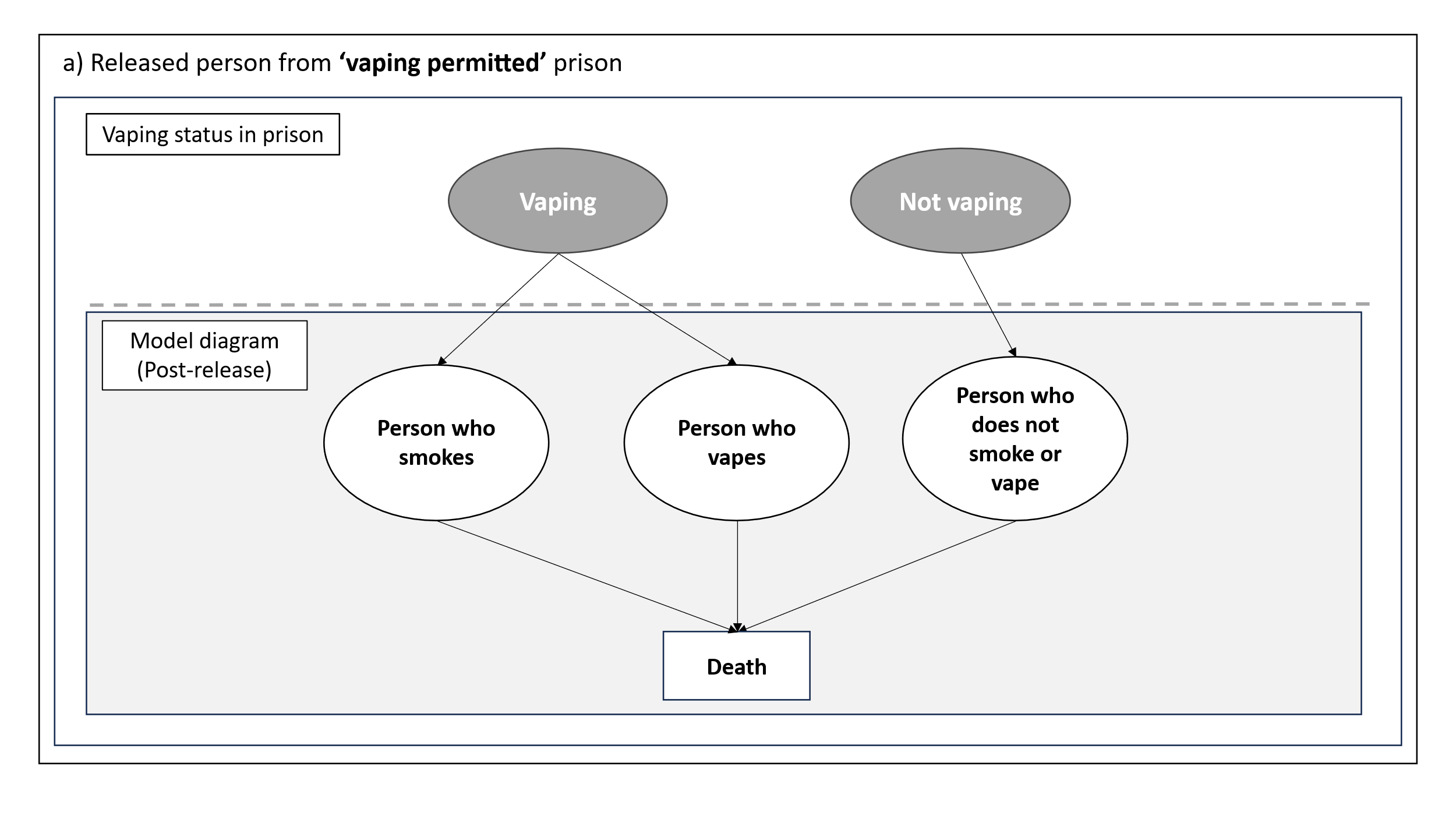

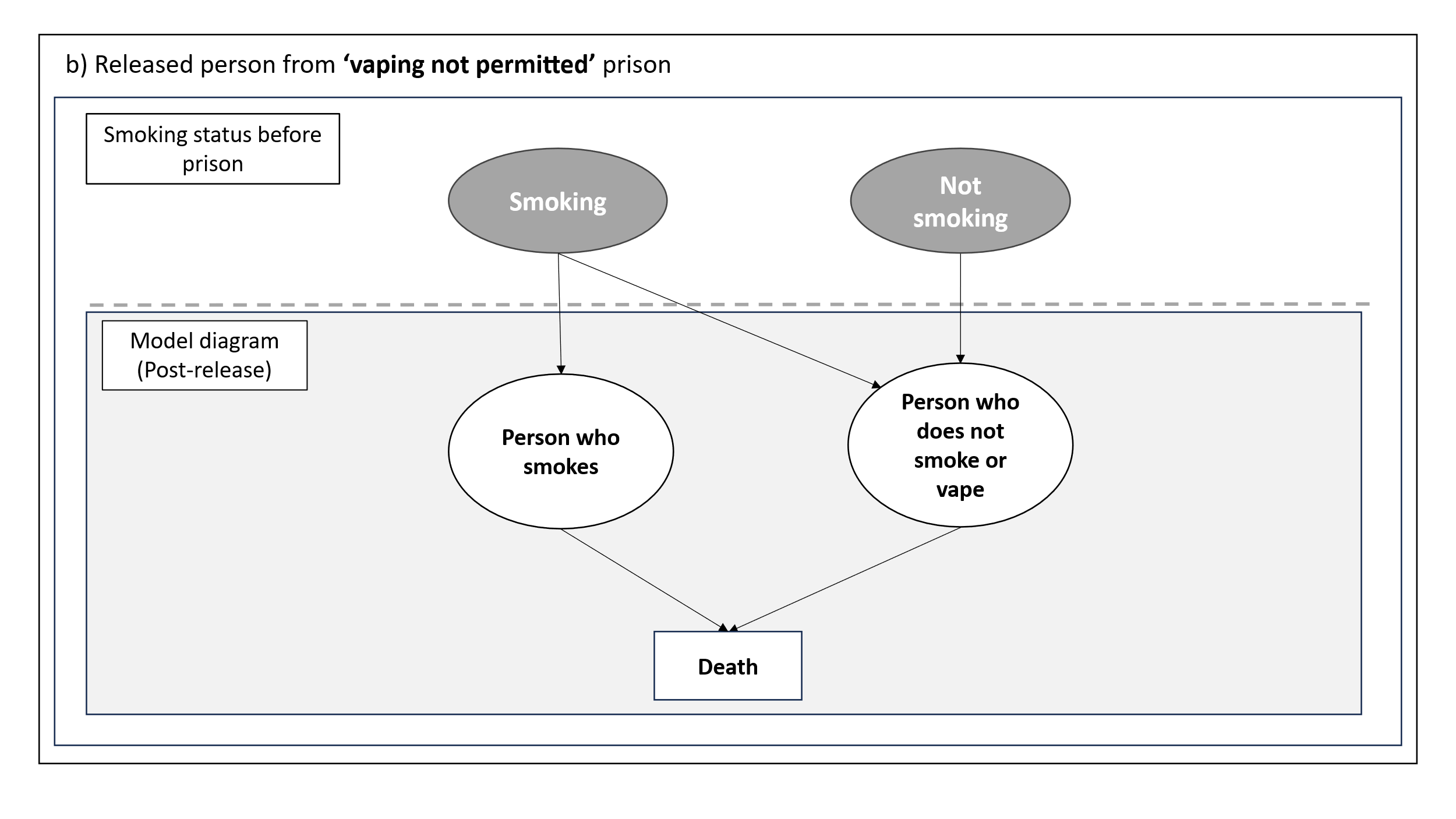


Figure 1 Released people model structure

### Partner/child model

For the partner/child model we assessed three scenarios; living with a released person who is a smoker, a vaper or is nicotine free (Figure 2).
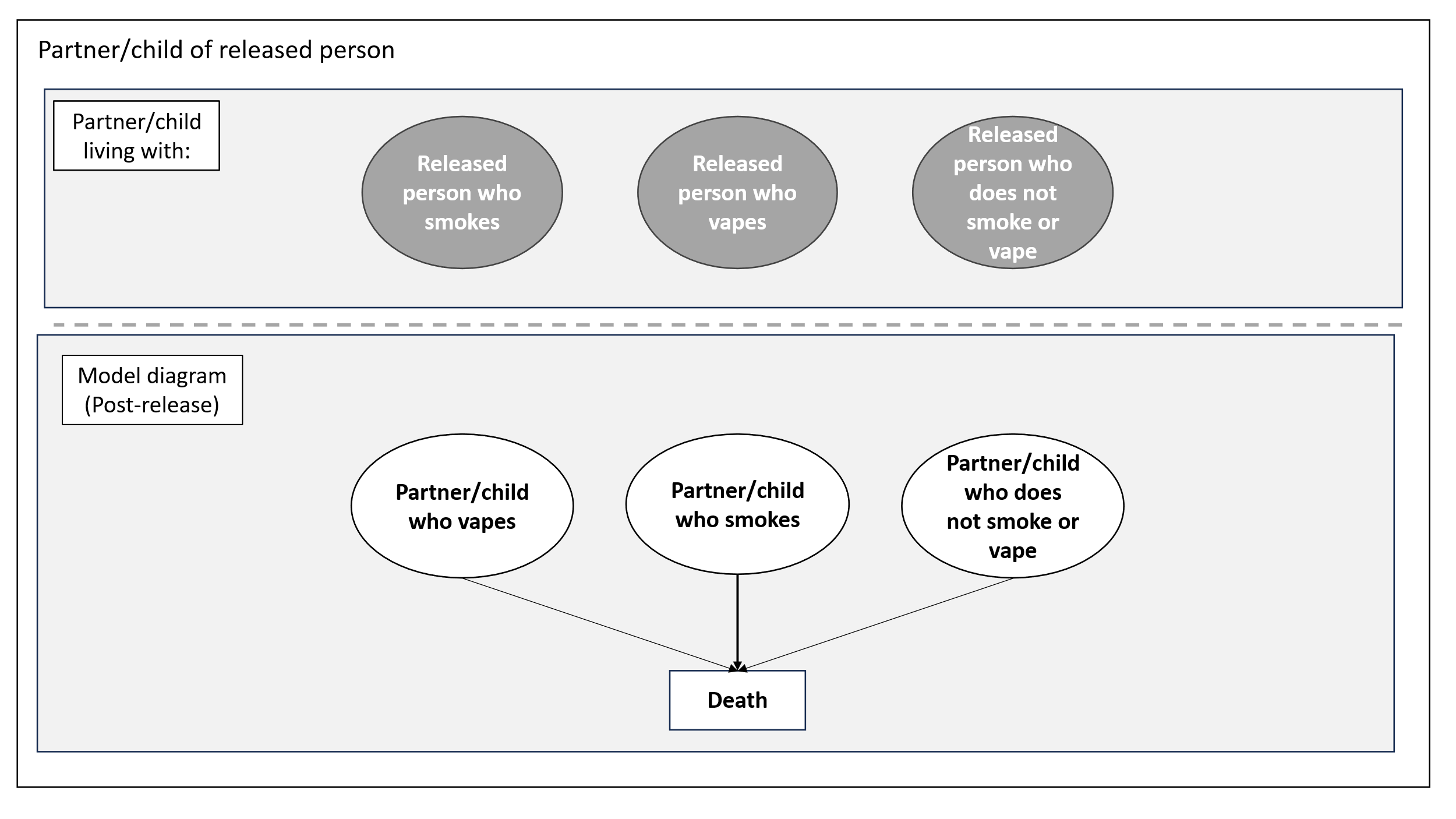


Figure 2 Model structure - partner and child

We applied general population smoking and vaping rates for women living in the most deprived neighbourhoods (from the Scottish Health Survey 2022) (4). We assumed that child uptake of smoking or vaping is dependent on the vaping or smoking status of the released person. An annual probability of smoking or vaping uptake is applied to children if the parent does not smoke(5). Children who live with a released person who smokes or vapes had an additional risk of smoking or vaping uptake applied(6).

Due to the lack of evidence on the proportion of released people who return to a family home with a partner and/or children, we have not included a link between the released people model and the partner/child models; results for partner/child models are presented separately. Making assumptions on the proportion of released people who would return to a family home is not possible due to this lack of evidence.

## Comparators

Due to limitations imposed by the pandemic and in the global evidence base, we assessed a hypothetical intervention, to support smoking cessation and prevent relapse, to smoking post-release (hereafter referred to as the ‘intervention’) in the model. We assumed that the intervention was available to people who smoked prior to imprisonment in the ‘no vaping permitted’ scenarios, and to people who vape in ‘vaping-permitted’ prisons, and offered around the time of release. The effectiveness of the intervention was applied in the model in the first year after release.

To model intervention impact we applied the costs and effectiveness of a medium intensity smoking cessation intervention reported in NICE smoking guidelines for the general population (7). We explored the impact of low and high intensity interventions on the results in sensitivity analyses. Low intensity consists of brief advice and self-help materials, medium intensity adds nicotine replacement therapy, and high intensity adds nicotine replacement therapy and specialist smoking cessation services (8).

We assumed that the proportion of the model cohort engaging in the intervention was equal to the number of people in prison reporting a desire to quit smoking in the 16^th^ SPS Prisoner Survey (9).

For released people we modelled four scenarios dependent on two factors: a) whether vaping is permitted in smokefree prisons, and b) whether the intervention is offered (Table 1).

Table 1 Model scenarios

| **Scenario 1**  Vaping permitted  No intervention | **Scenario 3**  Vaping permitted  Intervention offered |
| --- | --- |
| **Scenario 2**  No vaping permitted  No intervention | **Scenario 4**  No vaping permitted  Intervention offered |

To evaluate cost-effectiveness for released people we compared three pairs of the scenarios: i) vaping permitted in smokefree prisons vs no vaping permitted in smokefree prison with no intervention (1 vs 2); ii) no intervention vs intervention offered (vaping-permitted) (1 vs 3), and iii) no intervention vs intervention offered (no vaping permitted) (2 vs 4). These comparators were chosen to estimate costs and outcomes: i) between vaping-permitted and no vaping permitted in smokefree prisons after release with no support to quit smoking; ii) and iii) between offering an intervention and not offering an intervention in vaping and no vaping permitted smokefree prisons.

For partners and children we compared the costs and outcomes of living with released people who either smoke, vape or are nicotine free, allowing us to estimate outcomes of different smoking status’ as released people integrate back into their family units.

## Morbidity

Smoking related disease (SRD) prevalence (chronic obstructive pulmonary disease (COPD), coronary heart disease (CHD), lung cancer and stroke) was applied to all models (10), dependent on sex, age and smoking status. General population SRD prevalence was applied for nicotine-free people and people who vape (5). Additional age and sex specific relative risks were applied to model participants who currently or used to smoke (10).

There is emerging mixed evidence on vaping-related co-morbidities; after consultation with a chest physician, we applied acute bronchitis incidence to people who vape, which we consider to be a conservative approach (11, 12).

To account for SHS exposure, an increase in risk for CHD and lung cancer was applied to nicotine-free partners who live with a released person who smokes (13).

## Mortality

Mortality rates were applied to all models, dependent on sex, age and smoking status. Scottish general population mortality rates were applied to nicotine-free people and people who vape (14). Increased risk of mortality for people who currently smoke or used to smoke was applied (15), incorporating sex and age specific smoking prevalence for Scottish population(4).

A further adjustment was made for the known increased risk of mortality for released people (16-18). Finally, current evidence suggests that 1% of global mortality can be attributed to secondhand smoke (SHS) exposure; we incorporated this into our model for partners living with released people who smoke (13).

## Further model assumptions

SHS exposure is only applied to partners; we assume that children are only exposed to SHS for a short period before they no longer live with the released person (starting in the model at 15 years old).

For morbidity and mortality risks, released people are assumed to be male (Scottish Prisoner Survey reported 93% are male(9)), partners female, and children 50% male and 50% female. We acknowledge that not all released people will be male and not all partners will be female, this is a simplifying assumption to allow the model to be operationalised in a straightforward way.

The cost of bronchitis is based on reported spilt between inpatient and non-hospitalised treatment for COPD exacerbations, applying the cost of a mild to moderate exacerbation(19).

## Costs

The costs included in the evaluation include three types of resource use comprising: 1) healthcare use resulting from smoking related disease (SRD) and harms from vaping for all populations, and harms from being exposed to SHS (partners and children only); 2) personal (use smoking and vaping products), and 3) intervention update (released people only).

Unit costs were applied to resources, sourced from literature and published reports: bronchitis (19); SRD (20); personal nicotine products (21), and intervention (7). Unit costs are measured in GBP for 2021/22. Where relevant, costs were inflated using NHS cost inflation index (NHSCII) (22).

## Outcomes

Outcomes include quality adjusted life-years (QALYs), life-years (life expectancy) and prevalence of smoking/vaping/nicotine-free. Age-specific health utilities were taken from the literature for the general population to represent nicotine-free people and people who used to vape (23). A reduction in health utilities (disutility) was applied for people who smoke, vape, who used to smoke, and partners exposed to SHS. Disutilities were sourced from literature for people who smoke and used to smoke(24) and people who vape(25), and from the TIPs results for the impact of SHS (26). Disutilities for SRD were also applied (27).

Prevalence was extracted from the first cycle immediately after release.

## Table S1 Model input parameters

| **Parameter** | **Basecase value** | **Source** | **PSA distributions (alpha, beta)** | **Sensitivity analysis value** |
| --- | --- | --- | --- | --- |
| **Transition probabilities** | | | | |
| *Smoking status:* | | | | |
| Prevalence vaping in prison (relevant to vaping permitted comparators) | 76% | SPS canteen data (Cath Best analysis) | N/A | 60% SPS 17^th^ prisoner survey (2019) (9) |
| Tobacco smoking prevalence pre-prison (relevant to vaping not permitted comparators) | 68% | SPS 16^th^ prisoner survey (2017) (28) | N/A | N/A |
| Smoking status on release – vaping permitted comparators | 92% relapse to smoking (therefore assume 8% will continue vaping) | Jin et al 2021 (3) RCT - figure comes from control arm | N/A | 1) 100% relapse to smoking (0% continue to vape)  2) 50% relapse to smoking (50% continue to vape) |
| Smoking status on release - no vaping permitted comparators | 92% smokers (8% non-smokers/non-vapers) | Jin et al 2021 (3)  RCT - figure comes from control arm | N/A | 1) 94% Puljevic et al. (29) (6% non-smokers/non-vapers )  2) 86% Albany et al. (30) (14% non-smokers/non-vapers ) |
| Partner smoking status | Non-smoker/non vaper: 63%  Smoker: 21%  Former smoker: 6%  Vaper: 10% | Scottish Health Survey (2022) (4) | Beta (34.91, 20.5)  Beta (75.662, 284.632)  N/A  Beta (86.34, 777.02) | N/A |
| *Morbidity:* | | | | |
| Vapers lung injury (bronchitis) | 4.9% | Chaffee et al 2021 (11) | Beta (17.278, 335.33) | 1) 9.2%  2) 4.7% |
| Smoking related disease prevalence (COPD, CHD, lung cancer and stroke) | Various (age and sex dependent) | Jones et al 2018 (5) | N/A | N/A |
| Relative risks for smoking related diseases (COPD, CHD, lung cancer and stroke) | Various (age and smoking status dependent) | National Center for Chronic Disease Prevention and Health Promotion (US) Office on Smoking and Health 2014 (10) | Log normal (various) | N/A |
| Exposure to second-hand smoke (CHD and lung disease) | Various | Oberg et al (2011) (13) | Log normal (various) | N/A |
| *Mortality:* | | | | |
| Non-smoker and non-vaper | Various (age and sex dependent) | National records of Scotland (2022) (14) | N/A | N/A |
| Relative risk due to smoking status (former and current) | Various (age and smoking status dependent) | Doll et al (1994) & Scottish Health Survey (4, 15) | Log normal (various) | N/A |
| Exposure to second-hand smoke (CHD and lung disease) | 1% | Oberg et al (2011) (13) | N/A | N/A |
| Standardised mortality ratio – released people | 2.5 | Graham et al (2015) | N/A | N/A |
| **Health related utilities** | | | | |
| Non-smoker and ex-vaper | Various (age and sex dependent) | McNamara et al (2022) (23) | N/A | N/A |
| Non-smoker/non-vaper exposed to SHS (disutility) | -0.02 | McMeekin et al. (26) | Beta (94.10, 4610.9) | N/A |
| Tobacco smoker (disutility) | -0.062 | Maheswaran et al (2013) (24) | Beta (34.57, 522.96) | N/A |
| Former smoker (disutility) | -0.023 | Maheswaran et al (2013) (24) | Beta (27.46, 1166.3) | N/A |
| Smoking related disease (disutilities) | COPD: -0.1336  CHD: -0.0627  LC: -0.1192  Stroke -0.1171 | Sullivan et al (2011) (27) | Beta (30.96, 200.8)  Beta (21.41, 320.05)  Beta (6.65, 49.13)  Beta (82.57, 622.58) | N/A |
| Vaper (disutility) | -0.023 | Li et al (2020) (25) | N/A | N/A |
| Bronchitis | -0.0379 | NICE COPD NG114 (2018) (31) | Beta (6043.5, 153530) | N/A |
| **Costs** | | |  |  |
| E-cigarette spend | £600 annually | CRUK (2022) (21) | Gamma (88.51, 6.78) | N/A |
| Tobacco spend | £2,100 annually | CRUK (2022) (21) | Gamma (88.31,23.78) | N/A |
| Healthcare SRD | COPD: £930  CHD: £2,027  LC: £10,535  Stroke: £4,793 | Jones et al (2019) (5) | Gamma (99.99, 9.30)  Gamma (100, 20.27)  Gamma (100, 105.35)  Gamma (21583,0.22) | N/A |
| Vaping related lung disease | £257.08 | Asthma and lung UK (2023) (19) | N/A | N/A |
| **Intervention** | | | | |
| Intervention engaging | 53% tobacco smokers would like to give up smoking | 16th SPS prisoner survey (28) | N/A | 66% of smokers report wanting to give up 2022 SHS |
| Intervention effectiveness and cost | 6% quit smoking, cost £111.10 | NICE NG209 guideline (7) |  | 1) 4% quit smoking, cost £10.67  2) 15% quit smoking, cost £122.96 |
| **Miscellaneous** | | | | |
| Child uptake of smoking/vaping – parent non-smoker | 0.152 | Jones et al (2019) (5) | Beta (369.79, 2067.6) | 1) 0.2275  2) 0.0076 |
| RR child uptake of vaping (compared to parent non-smoker) | Parent ever vaper: 1.42  Parent ever smoker: 1.81 | Egger et al (2024) (6) | N/A | N/A |
| RR child uptake of smoking (compared to parent non-smoker) | Parent ever vaper: 1.97  Parent ever smoker: 2.59 | Egger et al (2024) (6) | N/A | N/A |
| Age released prisoner | 37 | SPS population statistics (2022/23) (32) | N/A | N/A |
| Age - partner | 37 | Assumption | N/A | N/A |
| Age - child | 15 | Assumption | N/A | N/A |
| Discount rate | 1.5% | NICE (2012) (1) | N/A | N/A |

CHD–coronary heart disease; chronic obstructive pulmonary disease–COPD; LC–lung cancer; SHS–second-hand smoke); SRD–smoking related disease

## Table S2 Sensitivity analysis results - Released people

| **Sensitivity analysis** | **Basecase** | **Permitting vaping v. not permitting vaping with no intervention in smokefree prison (Difference 1)** | | **Vaping permitted in smokefree prison (Difference 2)** | | **No vaping permitted in smokefree prison (Difference 3)** | |
| --- | --- | --- | --- | --- | --- | --- | --- |
|  |  | **Vaping permitted**  **(Scenario 1)** | **No vaping permitted (Scenario 2)** | **No intervention**  **(Scenario 1)** | **Intervention**  **(Scenario 3)** | **No vaping permitted (Scenario 2)** | **Intervention**  **(Scenario 4)** |
|  |  | **Incremental** | | | | | |
|  |  | **Costs** | **QALYs** | **Costs** | **QALYs** | **Costs** | **QALYs** |
|  |  | £3,764 (95% CI £2,715 to £5,125) | -0.252 (95% CI -0.355 to -0.17) | £1,022  (95% CI £866 to £1,209) | -0.044 (95% CI  -0.056 to -0.033) | £704 (95% CI  £561 to 871) | -0.037 (95% CI  -0.126 to 0.045) |
| Vaping status in prison 60% | 76% | -£2,143 (95% CI -£3,323 to -£920) | 0.209 (95% CI 0.108 to 0.294) | £816 (95% CI £698 to £948) | -0.035 (95% CI -0.044 to -0.025) | £714 (95% CI £581 to £874) | -0.039 (95% CI -0.124 to 0.044) |
| Vaping permitted: 100% smoking on release | 92% | £5,226 (95% CI £3,898 to £6,737 | -0.416 (95% CI -0.534 to -0.32 | £865 (95% CI £697 to £1,063 | -0.046 (95% CI -0.057 to -0.034) | £712 (95% CI £569 to £879) | -0.039 (95% CI -0.131 to 0.045) |
| Vaping permitted: 50% smoking on release |  | -£3,682 (95% CI -£5,936 to -£1,454) | 0.614 (95% CI 0.49 to 0.728) | £1,907(95% CI £1,754 to £2,076) | -0.036 (95% CI -0.047 to -0.024) | £708 (95% CI £562 to £867) | -0.041(95% CI -0.124 to 0.045) |
| No vaping permitted: 94% smoking on release (worst) | 92% | £3,229 (95% CI £2,299 to £4,658 | -0.208 (95% CI -0.315 to -0.135) | £1,023 (95% CI £862 to £1,201) | -0.044 (95% CI -0.055 to -0.032) | £720 (95% CI £570 to £878) | -0.039  (95% CI -0.122 to 0.04) |
| No vaping permitted: 86% smoking on release (best) |  | £5,374 (95% CI £4,177 to £6,870) | -0.377 (95% CI -0.477 to -0.29) | £1,029 (95% CI £874 to £1,200) | -0.044 (95% CI -0.055 to -0.031) | £664 (95% CI £526 to £806) | -0.036 (95% CI -0.117 to 0.047) |
| Vaping related lung disease 9.2% (worst) | 4.9% | £3,812 (95% CI £2,679 to £5,228) | -0.253 (95% CI -0.36 to -0.169) | £1,029 (95% CI (£873 to £1,206) | -0.044 (95% CI -0.056 to -0.032) | £709 (95% CI £564 to £871) | -0.037 (95% CI -0.12 to 0.05) |
| Vaping related lung disease 4.7% (best) |  | £3,781(95% CI £2,758 to £5,112) | -0.250 (95% CI -0.345 to -0.169) | £1,031(95% CI £880 to £1,201) | -0.044 (95% CI -0.056 to -0.032) | £712 (95% CI £576 to £869) | -0.036 (95% CI -0.119 to 0.048) |
| Intervention engaging 66% (best) | 53% | £3,761 (95% CI £2,740 to £5,044) | -0.249 (95% CI -0.341 to -0.173) | £1,225 (95% CI £1,019 to £1,439) | -0.055 (95% CI -0.069 to -0.039) | £882 (95% CI £700 to £1,078) | -0.046 (95% CI -0.128 to 0.037) |
| Intervention effectiveness and cost: low - 4% £10.67 | Mid - 6% and £111.10 | £3,766 (95% CI £2,718 to £5,188) | -0.248 (95% CI -0.354 to -0.171) | £784 (95% CI £681 to £897) | -0.030 (95% CI -0.038 to -0.021) | £495 (95% CI £401 to £596) | -0.026 (95% CI -0.103 to 0.057) |
| Intervention effectiveness: high – 15% £122.96 |  | £3,770 (95% CI £2,721 to £5,177) | -0.250 (95% CI -0.351 to -0.17 | £2,309 (95% CI £1,921 to £2,765) | -0.109 (95% CI -0.138 to -0.08 | £1,825 (95% CI £1,463 to £2,234) | -0.095 (95% CI -0.178 to -0.002 |

## Table S3 Sensitivity analysis results - Child

| **Uptake of smoking in children living with parent who smokes (basecase 15.2%)** | **Living with nicotine-free released prisoner** **(Scenario 1)** | | **Living with released prisoner who smokes** **(Scenario 2)** | | **Living with released prisoner who vapes** **(Scenario 3)** | |
| --- | --- | --- | --- | --- | --- | --- |
|  | **Mean** | **95% CI** | **Mean** | **95% CI** | **Mean** | **95% CI** |
| **Worst (22.8%)** | | | | | | |
| **Total costs** | £18,623 | £ 15,970 to £21,520 | £41,777 | £35,092 to £48,988 | £32,480 | £27,398 to £37,979 |
| **Life-years** | 25.62 | 25.58 to 25.65 | 25.32 | 25.23 to 25.40 | 25.44 | 25.37 to 25.50 |
| **QALYs** | 21.81 | 21.66 to 21.94 | 20.90 | 20.59 to 21.17 | 21.26 | 21.02 to 21.48 |
| **Best (7.6%)** | | | | | | |
| **Total costs** | £7,725 | £6,521 to £8,999 | £15,444 | £12,420 to £18,601 | £12,345 | £10,034 to £14,771 |
| **Life-years** | 25.75 | 25.73 to 25.76 | 25.65 | 25.60 to 25.68 | 25.69 | 25.65 to 25.71 |
| **QALYs** | 22.23 | 22.12 to 22.32 | 21.92 | 21.76 to 22.06 | 22.04 | 21.91 to 22.16 |
| **Prevalence** | | | | | | |
| **Basecase** | | | | | | |
| Non-nicotine | 70% |  | 33% |  | 49% |  |
| Non-nicotine (exposed to SHS | 0% |  | 0% |  | 0% |  |
| Person who smokes | 15% |  | 39% |  | 30% |  |
| Person who used to smoke | 0% |  | 0% |  | 0% |  |
| Person who vapes | 15% |  | 27% |  | 22% |  |
| Person who used to vape | 0% |  | 0% |  | 0% |  |
| **Worst** | | | | | | |
| Non-nicotine | 54% |  | 0% |  | 23% |  |
| Non-nicotine (exposed to SHS | 0% |  | 0% |  | 0% |  |
| Person who smokes | 23% |  | 59% |  | 45% |  |
| Person who used to smoke | 0% |  | 0% |  | 0% |  |
| Person who vapes | 23% |  | 41% |  | 32% |  |
| Person who used to vape | 0% |  | 0% |  | 0% |  |
| **Best** | | | | | | |
| Non-nicotine | 85% |  | 67% |  | 74% |  |
| Non-nicotine (exposed to SHS | 0% |  | 0% |  | 0% |  |
| Person who smokes | 8% |  | 20% |  | 15% |  |
| Person who used to smoke | 0% |  | 0% |  | 0% |  |
| Person who vapes | 8% |  | 14% |  | 11% |  |
| Person who used to vape | 0% |  | 0% |  | 0% |  |

## Cost-effectiveness plane – released people

The cost-effectiveness plane (Figure 3) visually illustrates results; the incremental costs and QALYs are plotted in four quadrants which show which comparators are more/less costly and beneficial.

Comparison 1 results (blue dots) show that if no intervention is offered, permitting vaping results in increased costs and decreased outcomes compared to not permitting vaping. Comparison 2 results (orange dots) show that after release from a vaping permitted prison not offering an intervention results in higher costs and lower outcomes compared to offering an intervention. Comparison 3 results (green dots) show that after release from a no vaping permitted prison, not offering an intervention results in higher costs compared to offering an intervention, but there is uncertainty whether offering an intervention is beneficial in terms of outcomes (the dots straddle the horizontal axis).


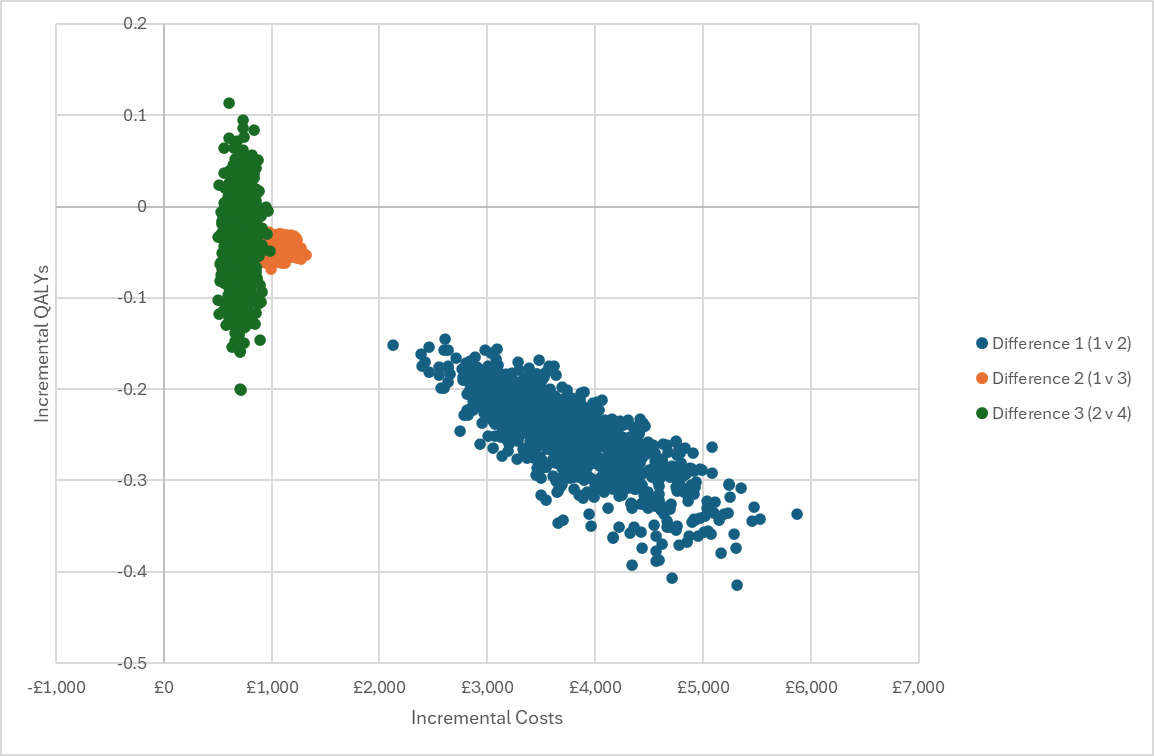


Figure 3 Cost-effectiveness plane - released people

## References

1. National Institute for Health and Care Excellence. Methods for the development of NICE public health guidance | 6 Incorporating health economics 2012 [Third edition:[Available from: <https://www.nice.org.uk/process/pmg4/chapter/incorporating-health-economics>.

2. Brown A, Woods-Brown C, Angus K, McMeekin N, Hunt K, Demou E. Recent evidence on rates and factors influencing smoking behaviours after release from smoke-free prisons: a scoping review. International Journal of Prison Health. 2024;20(4):450-65.

3. Jin X, Kinner SA, Hopkins R, Stockings E, Courtney RJ, Shakeshaft A, et al. Brief intervention on Smoking, Nutrition, Alcohol and Physical (SNAP) inactivity for smoking relapse prevention after release from smoke-free prisons: a study protocol for a multicentre, investigator-blinded, randomised controlled trial. BMJ open. 2018;8(10):e021326.

4. Scottish Government. The Scottish Health Survey 2022 [Available from: <https://www.gov.scot/publications/scottish-health-survey-2022-volume-1-main-report/pages/11/>.

5. Jones M, Smith M, Lewis S, Parrott S, Coleman T. A dynamic, modifiable model for estimating cost-effectiveness of smoking cessation interventions in pregnancy: application to an RCT of self-help delivered by text message. Addiction. 2019;114(2):353-65.

6. Egger S, Watts C, Dessaix A, Brooks A, Jenkinson E, Grogan P, et al. Parent's awareness of, and influence on, their 14-17-year-old child's vaping and smoking behaviours; an analysis of 3242 parent-child pairs in Australia. Addictive Behaviors. 2024;150.

7. National Institute for Health and Care Excellence. Tobacco: preventing uptake, promoting quitting and treating dependence, NICE guidance NG209 2023 [Available from: <https://www.nice.org.uk/guidance/ng209>.

8. Parrott S, Godfrey C, Raw M, West R, McNeill A. Guidance for commissioners on the cost effectiveness of smoking cessation interventions. Thorax. 1998;53:AS1-AS38.

9. Carnie J, Broderick R. Scottish Prison Service 17th Prisoner Survey 2019 [Available from: <https://www.sps.gov.uk/sites/default/files/2024-02/17thPrisonSurvey_2019_Research.pdf>.

10. National Center for Chronic Disease Prevention and Health Promotion (US) Office on Smoking and Health. The Health Consequences of Smoking - 50 Years of Progress: A Report of the Surgeon General. 2014.

11. Chaffee BW, Barrington-Trimis J, Liu F, Wu R, McConnell R, Krishnan-Sarin S, et al. E-cigarette use and adverse respiratory symptoms among adolescents and Young adults in the United States. Preventive Medicine. 2021;153.

12. Tackett AP, Urman R, Barrington-Trimis J, Liu F, Hong H, Pentz MA, et al. Prospective study of e-cigarette use and respiratory symptoms in adolescents and young adults. Thorax. 2024;79(2):163-8.

13. Oberg M, Jaakkola MS, Woodward A, Peruga A, Pruess-Ustuen A. Worldwide burden of disease from exposure to second-hand smoke: a retrospective analysis of data from 192 countries. Lancet. 2011;377(9760):139-46.

14. National Records of Scotland. Statistics and Data, Vital Events, Table 5.01(b) 2022 [Available from: <https://www.nrscotland.gov.uk/statistics-and-data/statistics/statistics-by-theme/vital-events/general-publications/vital-events-reference-tables/2022/list-of-data-tables#section5>.

15. Doll R, Peto R, Wheatley K, Gray R, Sutherland I. MORTALITY IN RELATION TO SMOKING - 40 YEARS OBSERVATIONS ON MALE BRITISH DOCTORS. British Medical Journal. 1994;309(6959):901-11.

16. Graham L, Fischbacher CM, Stockton D, Fraser A, Fleming M, Greig K. Understanding extreme mortality among prisoners: a national cohort study in Scotland using data linkage. European Journal of Public Health. 2015;25(5):879-85.

17. Spaulding AC, Eldridge GD, Chico CE, Morisseau N, Drobeniuc A, Fils-Aime R, et al. Smoking in Correctional Settings Worldwide: Prevalence, Bans, and Interventions. Epidemiologic Reviews. 2018;40(1):82-95.

18. Zlodre J, Fazel S. All-cause and external mortality in released prisoners: systematic review and meta-analysis. American journal of public health. 2012;102(12):e67-75.

19. Asthma and Lung UK. Investing in breath:  Measuring the economic cost of asthma and COPD in the UK and identifying ways to reduce it through better diagnosis and care 2023 [Available from: <https://www.asthmaandlung.org.uk/investing-breath-measuring-economic-cost-asthma-copd-uk-identifying-ways-reduce-it-through-better>.

20. Jones M, et al. Economics of Smoking in Pregnancy (ESIP) Model - The University of Nottingham 2019 [Available from: <https://www.nottingham.ac.uk/research/groups/tobaccoandalcohol/smoking-in-pregnancy/esip/index.aspx>.

21. Cancer Research UK. Is vaping harmful? 2022 [updated 2018-12-28. Available from: <https://www.cancerresearchuk.org/about-cancer/causes-of-cancer/smoking-and-cancer/is-vaping-harmful>.

22. Jones KC, Weatherly H, Birch S, Castelli A, Chalkley M, Dargan A, et al. *Unit Costs of Health and Social Care 2022 Manual*. Personal Social Services Research Unit (University of Kent) & Centre for Health Economics (University of York), Kent, UK 2023.

23. McNamara S, Schneider PP, Love-Koh J, Doran T, Gutacker N. Quality-Adjusted Life Expectancy Norms for the English Population. Value in Health. 2023;26(2):163-9.

24. Maheswaran H, Petrou S, Rees K, Stranges S. Estimating EQ-5D utility values for major health behavioural risk factors in England. Journal of Epidemiology and Community Health. 2013;67(2):172-80.

25. Li J, Hajek P, Pesola F, Wu Q, Phillips-Waller A, Przulj D, et al. Cost-effectiveness of e-cigarettes compared with nicotine replacement therapy in stop smoking services in England (TEC study): a randomized controlled trial. Addiction. 2020;115(3):507-17.

26. McMeekin N, Wu O, Boyd KA, Brown A, Tweed EJ, Best C, et al. Implementation of a national smoke-free prison policy: an economic evaluation within the Tobacco in Prisons (TIPs) study. Tobacco Control. 2023;32(6):701-8.

27. Sullivan PW, Slejko JF, Sculpher MJ, Ghushchyan V. Catalogue of EQ-5D Scores for the United Kingdom. Medical Decision Making. 2011;31(6):800-4.

28. Carnie J, Broderick R, Cameron J, Downie D, Williams G. Scottish Prison Service 16th Prisoner Survey 2017 [Available from: <https://www.sps.gov.uk/Corporate/Publications/Publication-6101.aspx>.

29. Puljevic C, de Andrade D, Coomber R, Kinner SA. Relapse to smoking following release from smoke-free correctional facilities in Queensland, Australia. Drug and Alcohol Dependence. 2018;187:127-33.

30. Albany H, Richmond R, Simpson M, Kariminia A, Hwang YI, Butler T. Smoking Beyond Prison Bans: The Impact of Prison Tobacco Bans on Smoking Among Prison Entrants. Journal of Correctional Health Care. 2021;27(4):280-8.

31. National Institute for Health and Care Excellence. Overview | Chronic obstructive pulmonary disease (acute exacerbation): antimicrobial prescribing | Guidance | NICE: NICE; 2024 [Available from: <https://www.nice.org.uk/guidance/ng114>.

32. Scottish Prison Service. Scottish Prison Population Statistics

2022-23 2023 [Available from: <https://www.gov.scot/binaries/content/documents/govscot/publications/statistics/2023/11/scottish-prison-population-statistics-2022-23/documents/report-22-23/report-22-23/govscot%3Adocument/Scottish%2BPrison%2BPopulation%2BStatistics%2B2022-23%2B-%2BAnalyt#:~:text=The%20overall%20prison%20population%20remained,in%202022%2D23%20was%207%2C426>.
